# Revisiting the DDE–Lactation Question: Association Not Confirmed in Breastfeeding Mothers

**Published:** 2008-02

**Authors:** Valerie J. Brown

Breastfeeding is known to be protective of newborn health, for example by lowering infant mortality and risk of infectious diseases. But breastfeeding is on the decline in some locales, especially in developing countries. Some studies have reported a link between elevated maternal serum DDE (the primary metabolite of the pesticide DDT) and shorter breastfeeding duration, suggesting that exposure to DDT affects the ability to breastfeed. New research does not confirm this hypothesis, however, and suggests possible ways to refine our understanding of the association previously reported with DDE **[*EHP* 116:179–183; Cupul-Uicab et al.]**.

Both DDT and DDE are retained in fatty tissue and excreted in breast milk. DDT was banned in the United States in 1972 but is still being used elsewhere to fight malaria. Given DDT’s prominence in malaria prevention, it is important to determine whether it affects infant and maternal health.

The current study involved 784 mother–son pairs from Tapachula, Chiapas, Mexico, where DDT had been used for about 40 years. The pairs had previously participated in a study of DDE’s antiandrogenic effects, in which the mothers’ serum levels of DDT and DDE were measured shortly after delivery. For the current study, the researchers interviewed each mother about every 2 months until her baby was weaned to determine the length of lactation.

The Tapachula women’s serum levels of DDE were about 15 times higher than recently measured levels in U.S. women. The team was not surprised to find higher DDE levels in first-time breastfeeders, because experienced breastfeeders would already have transferred some of their DDE body burden to earlier children. In the group as a whole, few women reported problems starting breastfeeding, and the median nursing duration was 10.8 months.

The researchers found a statistically significant positive association between DDE and shorter duration of lactation, but only among women who had previously breast-fed. This is consistent with some earlier research. But the team writes that it is probably an artifact rather than a causal link; otherwise, an association between DDE and shortened lactation would have been observed in both experienced and novice breastfeeders. Only 11 women could not breastfeed—too small a number for statistical significance—but these women did have higher median serum DDE concentrations than the other women in the study.

Although the authors found no link between DDE and shortened breastfeeding duration, they write that DDT exposure may make it more difficult to initiate breastfeeding, perhaps because of endocrine disruption early in lactation. For the first 2–3 days, lactation is controlled by hormones. If DDE behaves like estrogen, it could suppress initial milk production but have a weaker effect on established lactation. Future research could focus on this avenue by which DDE may affect successful breastfeeding.

## Figures and Tables

**Figure:**
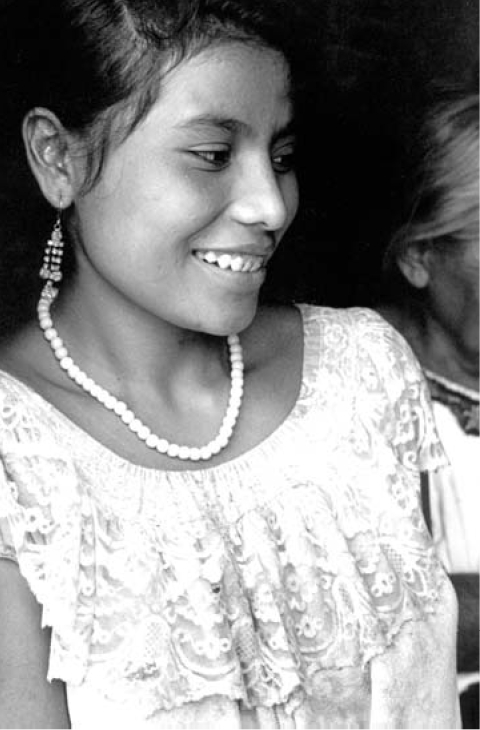
Good news for now Data from a study of Mexican mothers do not support earlier concerns that DDT exposure might impede lactation.

